# Email fraud: The search for psychological predictors of susceptibility

**DOI:** 10.1371/journal.pone.0209684

**Published:** 2019-01-16

**Authors:** Helen S. Jones, John N. Towse, Nicholas Race, Timothy Harrison

**Affiliations:** 1 Department of Psychology, Lancaster University, Lancaster, United Kingdom; 2 School of Computing and Communications, Lancaster University, Lancaster, United Kingdom; 3 Defence Science and Technology Laboratory, Salisbury, United Kingdom; University of Lleida, SPAIN

## Abstract

Decisions that we make about email legitimacy can result in a pernicious threat to security of both individuals and organisations. Yet user response to phishing emails is far from uniform; some respond while others do not. What is the source of this diversity in decision-making? From a psychological perspective, we consider cognitive and situational influences that might explain why certain users are more susceptible than others. Alongside an email judgment task employed as a proxy for fraud susceptibility, 224 participants completed a range of cognitive tasks. In addition, we manipulated time pressure for email legitimacy judgments. We identify cognitive reflection and sensation seeking as significant, albeit modest, predictors of susceptibility. Further to this, participants asked to make quicker responses made more judgment errors. We conclude there are cognitive signatures that partially contribute to email fraud susceptibility, with implications for efforts to limit online security breaches and train secure behaviors.

## Introduction

Phishing email fraud is a huge real-world problem—in terms of both the economic costs that can result from succumbing to it, as well as the organizational impact from attempting to contain it. Whilst computer science approaches have predominantly tried to block malicious attacks before they reach the user (e.g. [1, 2, 3]), these can often only help in the short term. Criminals circumvent these solutions, and fraudulent emails still reach users in large numbers every day. As users are essentially engaging in active decision-making about the trust or credibility of the email sender when deciding whether to respond, we address the need for a thorough exploration into the psychological constructs that may help to understand individual differences in susceptibility (as highlighted in [4]).

Jones, Towse, and Race [5] reviewed a disparate literature and articulated three different (potentially interrelated) theoretical psychological influences on email judgments. Firstly, the persuasiveness of the email message, such as its familiarity and the subtlety of fraud cues [6, 7, 8]. Secondly, the cognitive processing deployed in making legitimacy judgments, for example a reliance on more rational processing predicts lower trust in emails [9]. Finally, the theoretical influence of individual differences between users is considered, which is the main focus of the current research.

Insofar as analysis of individual differences in fraud susceptibility between users is relatively novel, there is no systematic literature specifying empirically established constructs already linked to email decision-making. In this paper, we will bring together a range of cognitive constructs that have not previously been linked in this context. In analyzing these constructs together, we can establish those that are more important to predicting susceptibility, and work towards building a cognitive profile of a more vulnerable user. From related fields there is reason to consider the predictive utility of several variables, which we explore further in the present paper. For example, self-control can predict fraud susceptibility across different media [10, 11], whilst the ‘big five’ personality factors may contribute some (but rather modest) influence on fraud victimization both online and off- [12, 13, 14]. Sensation seeking, as an assessment of inclination to engage in risk-taking behavior, has also been reported to predict compliance to phishing emails [15].

More broadly within individual differences and cognitive science, there is reason to also consider thinking style, in line with dual-system theories of reasoning (e.g. [16, 17, 18]). Such theories propose two psychological systems underpinning behavioral responses, the deployment of which depends on both the individual and the cognitive strain induced by a specific situation. System 1 relies on intuitive, immediate, and emotional responses to make decisions. This allows for rapid decision-making, with less time devoted to information processing, and thus this response type is usually dominant in decision-making scenarios. On the other hand, system 2 reasoning requires suppression of this initial intuitive response, in order to gather necessary information to allow hypothetical thinking. This more rational approach allows consideration of future consequences and allows a more considered decision to be made.

Individual differences in working memory capacity might affect the likelihood of resorting to system 1 processing [19, 20], which could in turn impair the identification of a deceptive email. These differences can occur both between subjects, but also within subjects, as influenced by a specific situation and the cognitive strain induced by it. Cognitive reflection [21] further considers such individual differences, by examining the information processing techniques employed by a person, either demonstrating impulsive decision-making or a reliance on more rational and contemplative approaches. This has previously been linked to every day consequences in online behavior, in particular the ability to recognize fake news [22]. Further, inhibition of intuitive responses [23] and a need for cognitive closure [24] may also demonstrate this ability to engage in more rational decision-making. The present paper therefore assesses the extent to which these individual difference variables together impact upon decision-making in the context of phishing emails.

In order to also explore the impact of thinking style within-subjects, the present paper considers the impact of time pressure on accurate email decision-making. Yan and Gozu [25] examined susceptibility to email fraud by distinguishing rational and intuitive decision-making conditions. Participants were told to either give rapid responses upon a first look at an email (intuitive), or told to take their time, and read the email carefully before deciding on their final response (rational). In the rational decision-making condition, participants correctly identified more emails as fraudulent. Further to this, research based on self-reports regarding the use of rational and intuitive decision-making strategies after receiving a simulated phishing email demonstrated that higher reliance on rational processing predicted lower trust in the legitimacy of the email [9]. In the present study, we develop a more controlled measure to assess the impact of explicit time constraints on decision-making accuracy regarding emails. In understanding such situational influences on susceptibility, it will be possible to build a picture of when users might be most at risk, and how we might focus efforts to protect them.

We developed a new email judgment task for the current study, in which participants were asked to judge the legitimacy of both phishing and legitimate email stimuli (rather than just phishing emails, as in [25]), before asking them to give legitimacy judgments on a six-point scale. This mirrors previous research (e.g. [26], [27]) that has also used a scale response method to emulate the range of response behaviors that a user may engage in, each of which carries with it a varying level of vulnerability. For example, simply opening the email itself carries a level of risk if embedded images are automatically downloaded, whilst clicking on the link within an email shows a greater level of vulnerability, but entering personal details on a linked web page demonstrates an even higher level. Since participants were asked to make a set of ordinal ratings on the email stimuli, we can report a range of analytic approaches derived from signal detection theory [28], providing complementary evidence to the normative metrics reported in this previous literature.

Although the lab-based methodology makes it difficult to assess each of these specific behaviors and may in fact inflate detection rates (see [29]), our focus is on variation between participants and attempting to capture the underlying behavioral patterns behind detection ability. Implementing a lab-based task also provides a controlled environment to assess psychological influences, whilst permitting the assessment of situational influences on decision-making.

In sum, we address three key aims concerning email decision-making from a psychological perspective. First, we quantify the difficulty of making email legitimacy judgments, assessing the extent to which individuals differ in accuracy across emails. Second, we explore potential psychological constructs and influences to explain these differences. Third, we seek to confirm the impact of time pressure on performance, as an indicator of the relevance of analytic, reflective and rational judgments for emails.

## Materials and methods

We report how we determined our sample size, all data exclusions, all manipulations, and all measures in the study [30]. This study was approved by the Lancaster University Research Ethics Committee, and the Ministry of Defence Research Ethics Committee. All participants gave fully informed written consent before taking part.

### Participants

We recruited 224 university students and staff (67 males and 156 females—in one case gender was not recorded) for this study. Sample size was based on power analysis for predicted effects based on previous literature, alongside cohort availability. Mean age was 19.37 (SD = 1.69), ranging from 18 to 30 years. All participants completed the email judgment task, which included a between-participants manipulation of time pressure. Due to time taken and potential fatigue effects (e.g. [31]), it was not feasible to administer all predictive cognitive tasks to all participants–therefore, we randomly assigned individuals to complete one set of psychological measures (either set 1, *N* = 120, or set 2, *N* = 101). A technical error led to incomplete predictive task data for three participants, so for this part of the study, *N* = 221.

### Tasks and materials

#### Email judgment task

Participants examined 36 emails (18 legitimate and 18 phishing emails–all stimuli are available as part of the supplementary materials), the majority of which were actual emails received by the research team, with a small number sourced online in blogs about phishing. The phishing and legitimate stimuli were matched where possible, by including examples from the same types of companies and with similar requests, such as resetting passwords or confirming transactions. In an initial pilot study, we asked 30 individuals to rate the legitimacy of the emails prior to the study, ensuring a good distribution of legitimacy ratings.

Emails were standardized, with recipient details and subject font kept constant. The content of the emails themselves, the subject wording, and the sender address were not modified. Participant instructions emphasized that each email should be considered relevant to the recipient and judged as such. Fifteen order permutations were randomly generated for the emails and varied across participants. For each email, participants made a legitimacy judgment on a 6-point scale, from ‘definitely phishing’ (1) to ‘definitely legitimate’ (6). To incentivize email performance, all participants were given the option to enter a cash prize draw for the three most accurate scores.

Participants were randomly assigned to receive one of two response speed instructions—they were told either that they only had five minutes to complete the entire email task (time pressure condition), or that they should complete the task at their own pace (no time pressure condition). Participants in the latter condition took around 10–15 minutes to respond to all stimuli, so we conclude that five minutes was plausibly a constraint on the amount of effortful cognition that participants were able to employ.

In order to analyze email detection behavior without the influence of response bias, signal detection analysis was employed. This encompassed analysis of both dichotomized phishing-legitimate response choices, as well as ordinal scale ratings of legitimacy. To dichotomize the data, each email decision was categorized as correct or incorrect, based on the scale responses given. Responses between 1 and 3 were taken as a response of ‘phishing’, whilst responses between 4 and 6 were taken as a response of ‘legitimate’. This allowed for a sum score of accurate responses to be generated, as well as for calculation of D-prime scores (d’) using formulae outlined in [32]. This uses the ‘hit’ rate (the proportion of phishing emails correctly identified) and the ‘false alarm’ rate (the proportion of legitimate emails incorrectly judged as phishing). A high, positive d’ score suggests a participant is discriminating well between the two stimuli types, whilst a low, negative number suggests they are poor at discriminating and show low accuracy.

For the scale data, individual receiver operating characteristics (ROC) curves were generated, from which the area under the curve (AUC) metric was calculated [33], which demonstrates participant ability to distinguish phishing from legitimate emails, unaffected by any bias in use of the scale. For the purpose of these calculations, scale responses were reversed so that a high number (i.e. 6) indicated strong belief the email was phishing, whilst a low number (i.e. 1) indicated belief that the email was legitimate. A maximum AUC statistic of 1 indicates perfect performance on the email task, with no bias, whilst a score between 0 and 0.5 indicates ineffective use of the scale or misunderstanding of the task [28].

#### Cognitive tasks

As noted above, participants completed one of two sets of tasks. Each set was of equivalent overall duration and comprised four measures, with both questionnaire and behavioral assessments across a spectrum of constructs. All questionnaires employed a 7-point Likert scale, allowing participants to give a neutral response (4). All measures were programmed and presented using PsychoPy [34].

**Fig 1:**
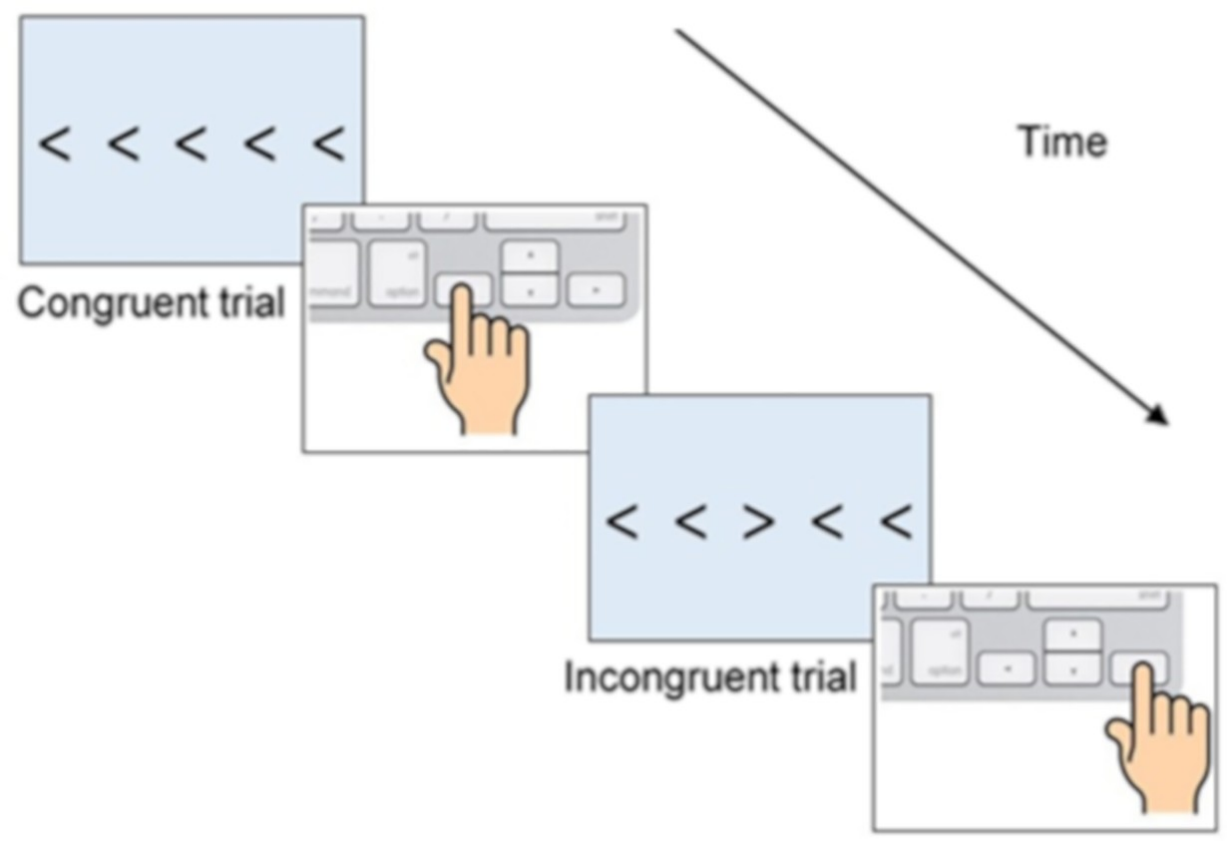
Flanker task–Stimulus shown on screen and correct participant response.

Set 1 assessed cognitive reflection, inhibition, the big five personality factors, and need for closure. The Cognitive Reflection Test (CRT; [21]) acts as a measure of impulsivity and cognitive reflection, consisting of three problems, each of which has an intuitive incorrect response. Each participant receives a score between 0 and 3, representing the sum of correct responses. The ability to inhibit distracting response cues was assessed with the Flanker task [35]. Participants responded as quickly as possible to the direction of the central arrow (left or right), which was either congruent, incongruent or neutral with respect to with the surrounding four arrows (See Fig 1 schematic). Neutral trials comprised the central arrow surrounded by four square shapes. Participants completed 24 practice trials followed by 192 trials over four blocks with an equal number of congruent, incongruent, and neutral trials.

The 50-item version of the IPIP (International Personality Item Pool; [36]) was used as a well-validated, shorter alternative to the original 100-item personality scale [37, 38]. This scale is based on the big-five personality factors (extraversion, agreeableness, conscientiousness, neuroticism, and intellect), with 10 items assessing each. After reverse scoring as appropriate, a sum score was obtained for each of the big-five personality factors. The 15 item ‘brief need for closure’ scale [39] is a shortened form of the original need for cognitive closure scale [40] and assesses a person’s desire to reach a conclusion to a problem as soon as possible. A mean score across all items generated a Need for Closure score.

Set 2 assessed inhibitory capacity, working memory capacity, self-control, and sensation-seeking. A behavioral index of inhibition involved a Stroop task [41]. Participants saw a color word on screen and responded to the font color—not the word itself. Following 27 practice trials, there were 144 trials, incorporating congruent (matching word and font color) and incongruent trials (mismatching word and font color). Font and word colors were red, green, and blue, with response keys r, g, and b respectively. Working memory capacity was assessed using a reading span task [42]. Participants read a set of independent sentences and afterwards recalled the last word from each. Participants also indicated after reading each sentence whether they thought it made sense or not; some sentences had the final word switched with another sentence to encourage comprehension rather than allowing participants to simply skip to the last word on each sentence. A typical administration format was followed varying list length across trial sets.

Summing responses to the 13-item brief self-control scale [43] produced a self-control score (after reverse scoring as appropriate). The brief sensation-seeking scale [44] consists of 8 items taken from the original 40-item scale [45] designed to capture risk-taking behavior. We calculated a mean score from the eight items.

### Procedure

Participants initially completed the email judgment task, with or without time pressure. In the former condition participants saw on-screen instructions, and were reminded by the researcher, that they only had five minutes to complete the task and so should work through the emails as quickly as possible. Those who were not in the time pressure condition were told to complete the task at their own pace. All participants saw task instructions, and information about the cash prizes available for those participants who performed best on the task.

Next, participants completed either task set 1 or 2. To avoid task order effects a Latin-square design was implemented for the cognitive tasks. Instructions were displayed for each task and, where appropriate, practice trials were provided.

## Results

Raw data and email task materials for this study are available as supplementary materials at [DOI: 10.17635/lancaster/researchdata/252 ].

### Email judgment task

As we had specifically designed the email task for the present study, we initially assessed patterns of detection ability. When calculating the AUC statistics, a number of participants (*N* = 9) were found to demonstrate response bias by favoring one end of the response scale, leading to degenerate AUC data. The AUC statistic for a further 9 participants were below .5, indicating poor use or misunderstanding of the scale. We excluded these participants from the AUC data, but note they were included in the analysis based on D-prime and accuracy scores, and all the underlying data are available in an independent repository.

Overall accuracy data indicates that average response accuracy lies at 68%, with no participant correctly identifying all 36 emails (*M* = 24.57, SD = 3.43), and only one participant correctly identifying all 18 phishing emails (*M* = 12.09, SD = 3.02). Across our measures of response behavior, statistics for Skewness (Number correct = -0.23, D-prime = 0.10, AUC = -0.25,) and Kurtosis (Number correct = -0.27, D-prime = -0.21, AUC = -0.46), indicated a reasonably normal distribution.

**Fig 2:**
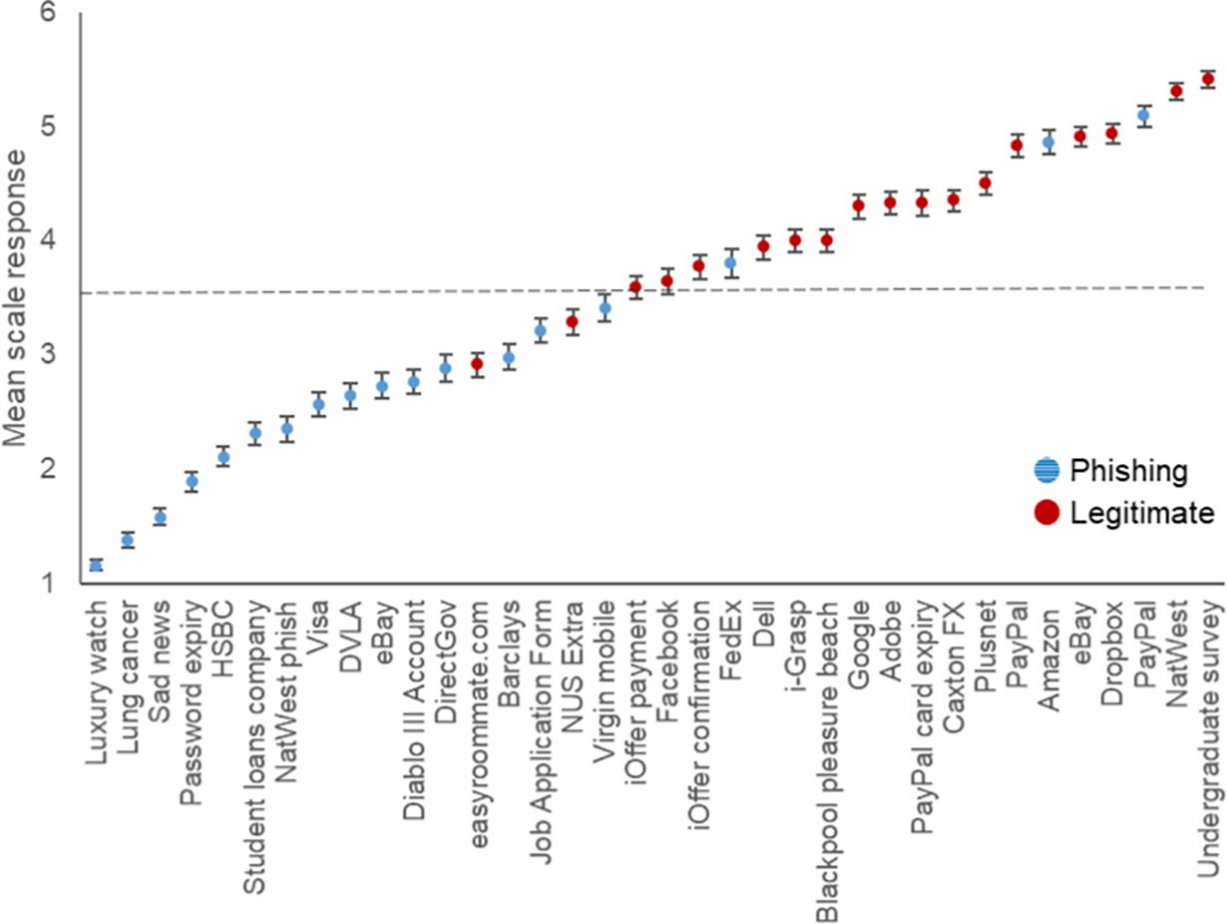
Mean rating (and standard error) across participants for each email stimulus (where 1 = definitely phishing and 6 = definitely legitimate).

Fig 2 describes performance on each email showing the average judgment across all 224 participants. Three phishing emails (FedEx, Amazon, and PayPal) led to mean response judgments that lie on the opposite side of the mid-point (as identified by the dashed line), indicating a predominance of incorrect responses. These emails were from well-known companies incorporating appropriately branded logos and formatting. Two legitimate emails (easyroommate.com and NUS Extra) also had mean scores on the incorrect side of the mid-point–these were poorly formatted emails with an unprofessional appearance implying that they were not genuine. Some emails had mean scores near to the extremes of the scale and very small standard deviation values, suggesting that they were easily identified as either phishing or legitimate. The spread of judgments reported in Fig 2 demonstrate that detection of fraud is far from a binary decision, and different message content elicits very different levels of confidence from the participants.

Table 1 reports the correlations between measures of email detection ability, demonstrating strong correlations between each of these (based on criteria from Cohen [46], suggesting *r* > .5 determines a strong relationship).

**Table 1:** Correlations between measures from the email legitimacy task.

	1	2	3
1. No. correct	1.00		
2. D-prime	.97**	1.00	
3. AUC	.81**	.81**	1.00

Note.

**p < .01 (two-tailed)

A one-way ANOVA demonstrated that D-prime scores for participants in the time pressure condition (*M* = 1.00, *SD* = 0.56) showed lower discriminative sensitivity (i.e. poorer performance) in phishing detection that those without time pressure (*M* = 1.19, *SD* = 0.62), *F* (1, 222) = 5.66, *p* < .05, η^2^ = .03. A significant difference was also obtained using the AUC estimates *(M* = .73, *SD* = 0.10 vs. *M* = .76, *SD* = 0.10 for time pressure vs. no time pressure conditions), *F* (1, 204) = 8.24, *p* < .01, η^2^ = .04.

### Predictive measures—Set 1

In the cognitive reflection test, there were eight cases of data failure (*N* = 112). Performance on the Flanker task is based on the mean difference between (correct) response time on congruent and incongruent trials. When testing began, a technical error meant that data was not collected for the Flanker task for the first 18 participants. In addition, one further case was removed from this analysis—an extreme outlier compared with other participants’ response times (*z* = -8.17) (*N* = 101). Descriptive statistics and Cronbach’s alpha coefficients for relevant measures are shown in Table 2. Additionally, correlations between measures and the email legitimacy task are shown in Table 3. These correlations point to a positive association between CRT scores and email judgments.

**Table 2:** Descriptive statistics for cognitive tasks.

Cognitive measure	N	Mean	SD	α
Set 1				
Cognitive reflection test	112	1.22	1.18	-
IPIP				
Extraversion	120	32.35	7.67	.86
Agreeableness	120	40.58	6.00	.82
Conscientiousness	120	33.87	6.54	.79
Neuroticism	120	29.62	8.66	.86
Intellect	120	36.03	5.29	.74
Flanker test	101	4.58	104.77	-
Need for closure scale	120	3.13	0.55	.79
Set 2				
Self-control	99	38.87	8.25	.83
Sensation seeking	99	3.20	0.74	.72
Stroop task	98	81.82	74.66	-
Reading span	101	2.23	1.52	-

**Table 3:** Correlations between cognitive tasks in set 1 and email legitimacy task scores.

	1	2	3	4	5	6	7	8	9
1. D-prime	1.00								
2. AUC	.81**	1.00							
3. CRT	.26**	.27**	1.00						
4. Extraversion	-.13	-.07	-.07	1.00					
5. Agreeableness	-.17	-.14	-.27**	.31**	1.00				
6. Conscientiousness	-.07	-.02	-.14	.01	.18*	1.00			
7. Neuroticism	.06	.07	.06	.18	-.02	.07	1.00		
8. Intellect	.00	.08	.03	.12	.12	.20*	.07	1.00	
9. Flanker	—.14	—.13	-.12	.17	—.12	-.01	-.09	—.02	1.00
10. Need for closure	-.06	-.08	-.10	-.27	-.15	.23*	-.30**	-.03	-.02

**p < .01

*p < .05 (two-tailed)

Stepwise multiple regression analysis assessed whether psychological measures predicted accuracy in email judgments. The results, shown in Table 4, demonstrate that the cognitive reflection test was a significant predictor based on both D-prime scores and AUC estimates as measures of sensitivity. All other measures were found not to be significant predictors.

**Table 4:** Stepwise regression for set 1.

Model	R^2^	Adjusted R^2^	β	F	*p*
D-prime					
CRT	.09	.08	0.30**	9.01	< .01
AUC					
CRT	.08	.06	0.27*	6.86	.01

Note.

**p < .01

*p < .05 (two-tailed).

β values are standardized.

### Set 2

As with the Flanker task, the Stroop task was based on the mean difference between congruent and incongruent response times for correct trials. Two cases were removed from this analysis as extreme outliers compared with other participants’ response times (*z* > 3.00) (*N* = 98). Reading span was calculated based on the highest sequence of sentence-end words correctly recalled. For example, if a participant was able to completely recall two out of three sets of four words or more, but were unable to when shown five words at a time, then their span size would be four. A technical error made it impossible to distinguish participants who reached span size five or six because the last set of memoranda were not recorded, so all participants reaching this point were recorded as having span size five. As only five percent of participants reached at least span size five, this score-coarseness is unlikely to be a major issue.

Table 2 reports descriptive statistics and Cronbach’s alpha coefficients for relevant measures, while Table 5 reports correlations between the tasks and email judgments. These correlations show a significant negative relationship between D-prime scores and sensation-seeking.

**Table 5:** Correlations between cognitive tasks in set 2 and email legitimacy task performance.

	1	2	3	4	5	6
1. D-prime	1.00					
2. AUC	.80**	1.00				
3. Self-control score	.08	-.01	1.00			
4. Sensation-seeking score	-.21*	-.14	-.11	1.00		
5. Stroop test	-.00	-.04	-.00	-.08	1.00	
6. Reading span	.01	.04	.08	.08	-.07	1.00

Note.

**p < .01

*p < .05 (two-tailed)

Again, stepwise multiple regression analysis investigated which variables best predict email performance. When D-prime was taken as the dependent variable, the best-fit model included sensation seeking (β = -.21, *p* < .05) as a significant predictor. This model produced an R^2^ value of .04 (adjusted R^2^ = .03, *F* (1, 97) = 4.41, *p* < .05). This indicates that participants who demonstrated higher sensation seeking were poor at discriminating between phishing and legitimate stimuli. However, when AUC statistics were taken as the dependent variable, no significant predictors were found.

## Discussion

We set out to address three research aims regarding email decision-making. First, to explore the extent to which accuracy in email response decisions varies between participants. Email decisions overall were highly error prone–no participant answered all questions correctly and mean performance was 68%. In practical settings, of course, misclassification of even a single email can result in fraud victimization. Based on the average response across all participants, we identify phishing emails that are designed with a high level of consistency and purporting to come from well-known companies, likely to be familiar to most participants (e.g. PayPal), as the most convincing.

Our second aim was to explore the psychological constructs that may explain this variability in participant accuracy. We found evidence with signal detection theory measures that sensation seeking and cognitive reflection were modest predictors of the ability to discriminate email messages. Other measures, notably perhaps personality constructs given prior reports in the literature, were not systematically linked to performance. In other words, data offer evidence that there are psychological markers (specifically, cognitive as well as social influences) of vulnerability to making erroneous email decisions—though this provides only a partial explanation of performance. At the same time, our data offer valuable insight into the constructs that are less relevant to susceptibility, indicating where preventative resources should be focused and where these may be less effective.

Previous research linking sensation seeking and risky decision-making [44] suggested that higher sensation seeking scores would correlate with erroneous decision-making in email management, and our results confirm this based on the dichotomous data. This suggests that whilst ability to discriminate between the phishing and legitimate stimuli may be impaired by sensation seeking, this impairment is not reflected in the confidence scores that participants gave. It may be argued that at the point users respond to phishing they are not necessarily demonstrating risk-taking behavior insofar as they believe that an email is legitimate or genuine (i.e. riskiness may be an attribution given with hindsight). Impulsivity may pose a more viable mediating construct between sensation seeking and susceptibility. In other words, participants demonstrating higher impulsivity (through the measure of sensation seeking) make more erroneous judgments. This would be consistent with a viewpoint that impulsivity may shape the likelihood of an intuitive rather than systematic response, regardless of the email specific consequences.

Performance on the CRT also predicted susceptibility. Data do not unequivocally specify the processes responsible for this, yet this finding is consistent with dual-system processing theories (e.g. [16, 17, 18, 47]) in email decision-making. Cognitive reflection may be linked to a preference for an intuitive processing style, or relatively impaired inhibition efficiency indicative of system 1 processing. Those participants with lower cognitive reflection may have been more reliant on immediate, intuitive responses during the decision-making process, which can lead to more erroneous judgments due to missed information [23].

That our data suggest a number of psychological constructs do *not* systematically contribute to email response behavior is also noteworthy. This is especially intriguing in relation to factors that have previously been associated with susceptibility, such as personality ([12, 13] –although the effects are very small in this dataset, [14]) and self-control [10]. Whilst different methodologies were used to assess fraud susceptibility, victimization, or the comparison group in these studies, we expected to see a replication of previous findings. As well as pointing to the importance of establishing the link between methodological approach and outcome, these null results provide additional complimentary evidence to inform future research–for example our data suggest that psychological constructs other than personality play an important role. In this way, data point to the value of widening the search for psychological markers beyond the generic personality construct.

In both task sets, we recognize the proportion of variance explained by the regression models was modest. First, this strongly suggests that, even assuming the variables in each set offer independent contributions, there is much more to explaining susceptibility than these constructs alone. In this sense, it is also possible that alternative factors *jointly* influence susceptibility–in particular email content and situation. This study is just a starting point towards a full characterization of psychological influences. Second, there are of course constructs we have not had the opportunity to explore here, which may be relevant and independent. Third, it is important to interpret the current data bearing in mind some influences may be masked or suppressed statistically as a result of our choice of measures—but even here the null findings also provide an important insight about those that are *not* systematically relevant to susceptibility. In this context, the importance of minimizing measurement error in both predicted and predictor constructs is apparent. Therefore, the true relationships between constructs may be shown to be stronger than reported here when better and more sensitive measures can be derived from a wider body of convergent research.

Finally, we suggest there may be an indeterminate component to performance—fraud victimization has a probabilistic component. In other words, it simply wouldn’t be plausible to expect a susceptible individual to display their vulnerability on *every* email judgment. Just as no individual correctly categorized every email in our test set (i.e. everyone shows some vulnerability), no individual incorrectly judged all emails either. Consequently, it is vital for the research field to recognize that judgements provide noisy signals, which potentially masks the strength of relationships.

This leads on to the third aim of this research, which was to consider the impact of time pressure on email judgment accuracy, as a situational influence on performance. We confirmed that participants under time pressure (vs without such pressure) made fewer correct decisions, replicating Yan & Gozu [25]. Again, this may be linked to dual-system theories of reasoning, demonstrating a reliance on more intuitive, system 1 based processing of email stimuli when under increased time pressure. Those in the no time pressure condition may have been more likely to employ rational decision-making mechanisms, enabling them to process additional information and cues within the email stimuli that were missed by those who were under time pressure. Whilst significant, the effect size is relatively small, implying only a modest influence under the specified constraints (of course we cannot know how this effect might change with more severe time constraints). Also, the effect may vary between emails, with some requiring more consideration and contemplation than others. At this stage, we have only aggregated the effect across stimuli. Data thus offer some support for campaigns such as “Take Five” (a UK Government campaign encouraging users to stop and think before making decisions around personal data and financial information; https://takefive-stopfraud.org.uk), whilst at the same time emphasizing that this may be only a small part of the picture.

We believe this study offers a highly important contribution to the literature, identifying some of the psychological constructs that contribute to email fraud susceptibility. In line with recommendations for further research in this area from Williams et al. [4] and Wood et al. [48], this paper offers the first comprehensive assessment of a wide range of constructs in an effort to establish a cognitive profile of susceptibility. A theoretical model outlined by Jones et al. [5] based on the existing literature integrates three core elements as explanations of susceptibility to a phishing email in the moment—cognitive processing as a result of situational factors, individual differences between users, and persuasiveness of email content. Each of these is reinforced in the data reported in this paper. Time pressure represents a situational factor that is shown to impair decision accuracy, undoubtedly just one of many such factors in our daily environment. Individual differences are substantial, and at least partially explicable, here in terms of sensation seeking and cognitive reflection. Moreover, error rates are quite consistent with the argument that message persuasiveness reflects content such as sender familiarity and consistency.

### Limitations and future research

There remain several key research questions or issues highlighted by the present work. Convergent evidence on the validity of the email task methodology with respect to real-world susceptibility, for example (see also [49]), along with further analysis of additional psychological constructs using alternative measures of susceptibility and consideration of specific persuasive techniques employed in email content (e.g. authority [50]) may also offer complementary perspectives to those discussed here. Whilst evidence across measures points to the role of inhibitory capacity and impulsivity in email detection accuracy, some constructs measured were *not* significant predictors of susceptibility; it is not clear at this stage whether they are simply not relevant, or whether their contribution may be masked by other variables. It should also be noted that participants only completed one set of tasks, and so findings around individual difference factors are only applicable to those participants who completed the same subset rather than the whole sample. This was due to practical and ethical constraints that limited the number of tasks participants could plausibly complete without being impacted by fatigue and attentional issues.

We have not replicated previous findings in all cases—for example we do not find evidence for a systematic contribution from personality or self-control in email fraud susceptibility, or at least we show that any such impact is rather small. This in turn emphasizes the importance of consistency across research in measurement of susceptibility. The email task employed in the current study is a lab-based method requiring participants to actively differentiate between phishing and legitimate emails, which may exaggerate accuracy in decision-making (see [29]). This is not necessarily representative of real world email management behavior, where users *interact* with their inbox, are familiar with those individuals or companies they usually have contact with and will usually know whether an email is relevant to them. On the other hand, the task provides an ethically relatively straightforward measure which has been reported by other researchers, but with different stimuli. The development of a universal and well-tested measure, in which email stimuli are also carefully matched on length, inclusion of logos, and measurement of detection would provide a good starting point for comparable research outputs. Finally, we implemented a six-point ordinal scale measure of the email stimuli, to capture degrees of certainty or uncertainty about the stimuli. However, we recognize that this also introduces potential individual differences in participants’ interpretation and use of this scale—in particular, with regards to the threshold for rating message legitimacy. The way in which this threshold reflects real world email behavior may therefore not be consistently reflected in the way different participants use this scale measure.

## Conclusions

In conclusion, we suggest the principal contribution of the current study is to offer grounding for research into the individual differences between users in email decision-making. It starts to characterize the cognitive profile of those who are most at risk, emphasizing the importance of inhibitory capacity and impulsivity in the decision-making process. Alongside this, other factors are highlighted that may be relevant, such as time pressure, and the content of the emails themselves. Thus, our research provides a conceptual insight into a very current problem, with opportunity to use psychological data to optimize training or intervention programs to address this persistent security threat at the point that an email reaches the user.
